# Supplementary figures and images for: The Endosperm-Specific Gene OsEnS-42 Regulates Seed Vigor and Grain Quality
Source: Plants (Basel). 2025 Aug 11;14(16):2492. doi: 10.3390/plants14162492 (PMC12389547; doi:10.3390/plants14162492)

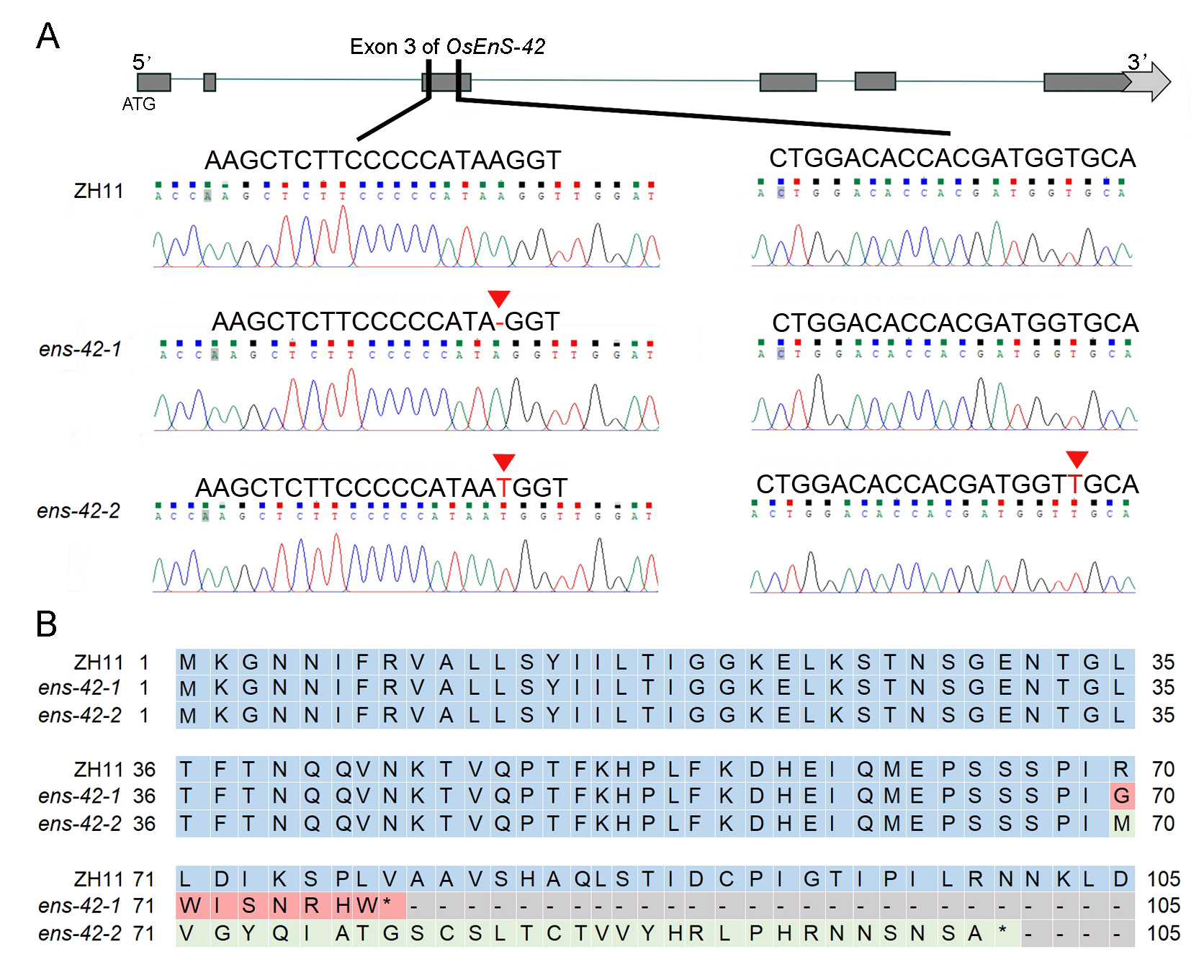

Supplement: Supplementary file 1 [file plants-14-02492-s001.zip › Figure S1.-revised.jpg]

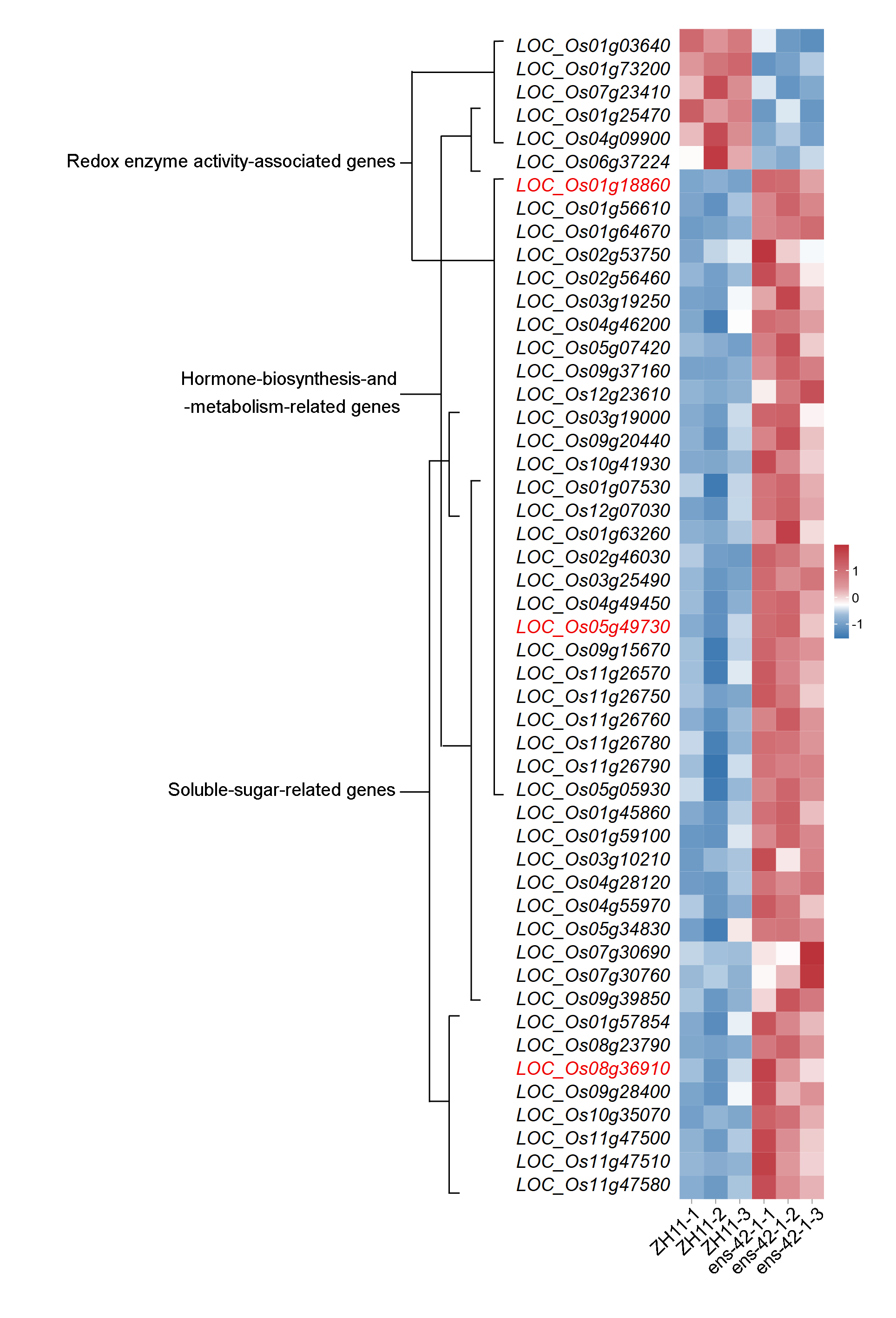

Supplement: Supplementary file 1 [file plants-14-02492-s001.zip › Figure S2-revised.jpg]

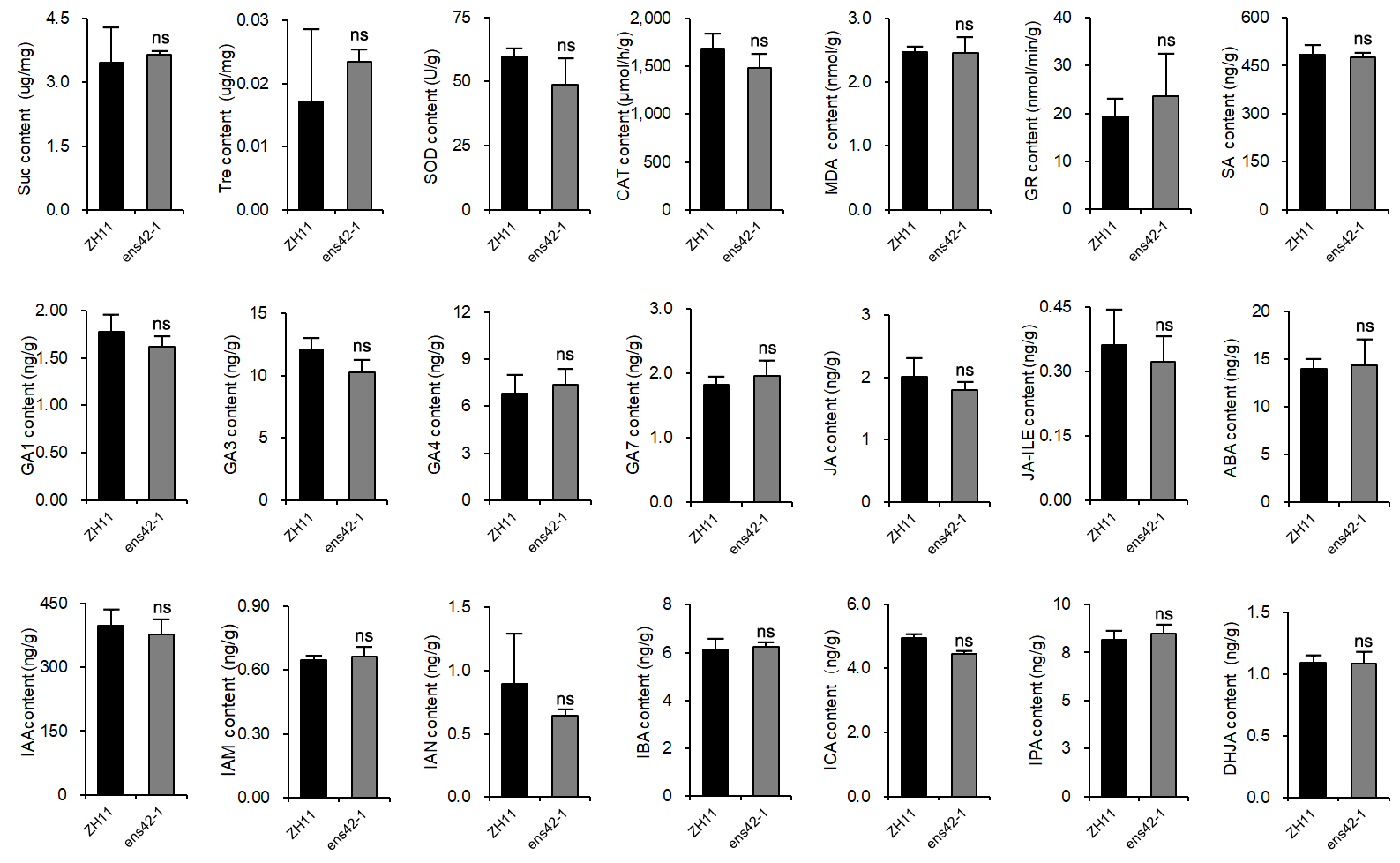

Supplement: Supplementary file 1 [file plants-14-02492-s001.zip › Figure S3.jpg]

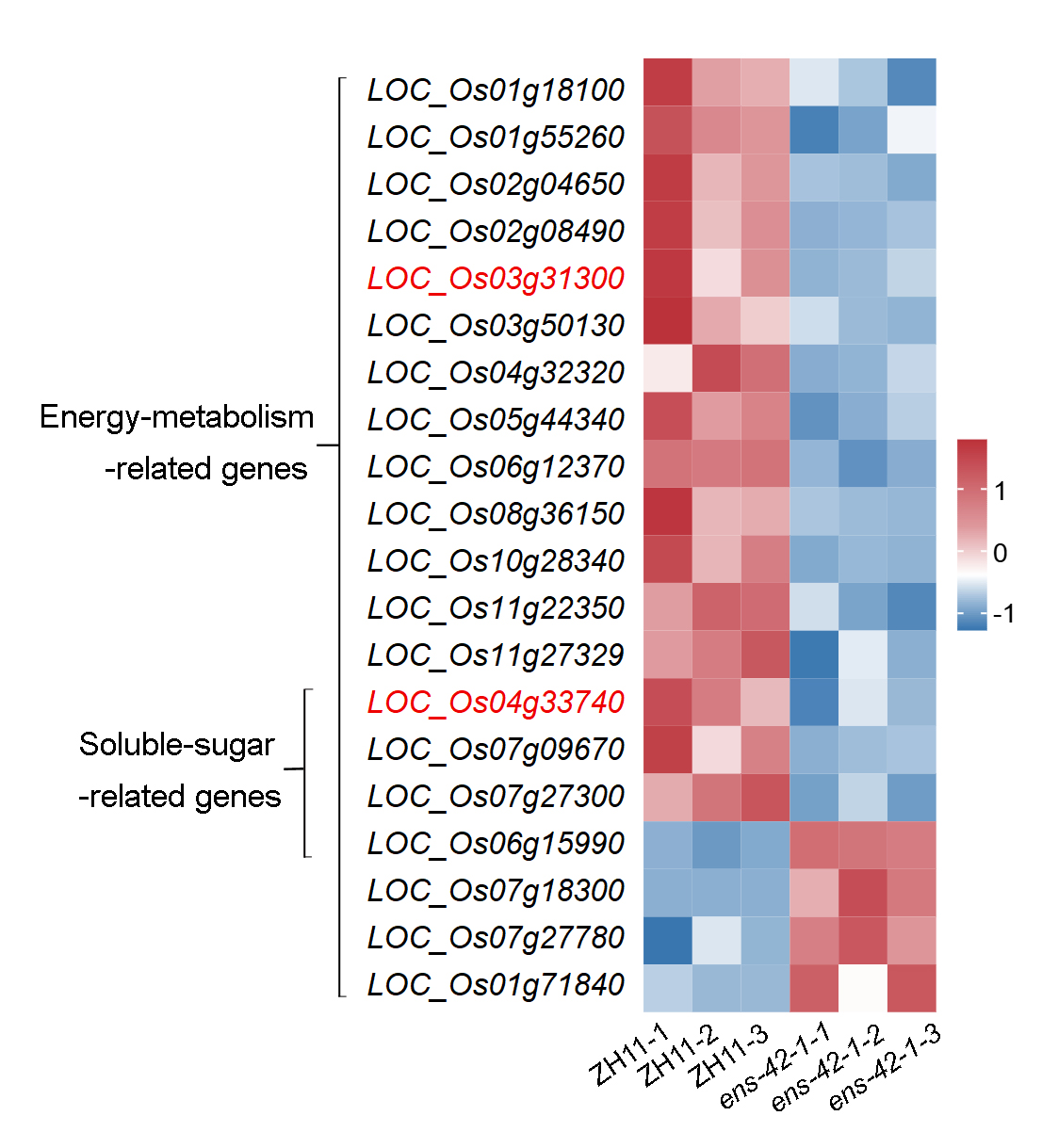

Supplement: Supplementary file 1 [file plants-14-02492-s001.zip › Figure S4.jpg]
